# Adaptation responses to salt stress in the gut of *Poecilia reticulata*

**DOI:** 10.1080/19768354.2025.2451413

**Published:** 2025-01-18

**Authors:** Hyerim Lee, Hyunjae Yeo, Jihye Park, Keunsoo Kang, Sun-Ju Yi, Kyunghwan Kim

**Affiliations:** a Department of Biological Sciences and Biotechnology, Chungbuk National University, Cheongju, Republic of Korea; b Department of Microbiology, Dankook University, Cheonan, Republic of Korea

**Keywords:** *Poecilia reticulata*, salt stress, osmoregulation, intestine, transcriptome

## Abstract

Osmoregulation is essential for the survival of aquatic organisms, particularly teleost fish facing osmotic challenges in environments characterized by variable salinity. While the gills are known for ion exchange, the intestine’s role in water and salt absorption is gaining attention. Here, we investigated the adaptive responses of the intestine to salinity stress in guppies (*Poecilia reticulata*), observing significant morphological and transcriptomic alterations. Guppies showed superior salt tolerance compared to zebrafish (*Danio rerio*). Increasing salinity reduced villus length and intestinal diameter in guppies, while zebrafish exhibited damage to villus structure and loss of goblet cells. Transcriptomic analysis identified key genes involved in osmoregulation, tissue remodeling, and immune modulation. Upregulated genes included the solute carrier transporters *slc2al* and *slc3al*, which facilitate ion and water transport, as well as a transcription factor AP-1 subunit and phosphatidylinositol-4,5-bisphosphate 3-kinase catalytic subunit beta, both of which participate in tissue repair and growth responses. In contrast, many genes related to the innate immune system (such as *Tnfaip6*) were downregulated, suggesting a shift toward the prioritization of osmoregulatory functions over immune responses. Interestingly, the differential expression of adaptation genes was linked to variations in epigenetic modifications and transcription factor activity. Transcription factors crucial for adapting to salt stress, such as *bhlhe40*, *cebpd*, and *gata6,* were progressively upregulated in guppies but remained downregulated in zebrafish. Our findings highlight the intricate mechanisms of adaptation to salinity stress in *P. reticulata*, providing insights into osmoregulatory mechanisms involving the intestine in aquatic organisms.

## Introduction

Diverse environmental cues affect different aspects of fish biology that are mediated by epigenetic mechanisms, including DNA methylation, histone modification, and non-coding RNAs, resulting in changes in gene expression. Epigenetic alterations contribute to fish adaptation in response to environmental stressors, which can be represented by changes in key parameters such as temperature, salinity, nutrients, dissolved oxygen, and pH. In the process of adaptation to these stressors, the alteration of DNA methylation is the most frequently reported type of epigenetic regulation (Shen et al. 2023).

The guppy (*Poecilia reticulata*) a small euryhaline fish inhabiting freshwater environments, serves as an excellent model organism for studying rapid adaptation due to its short life cycle and remarkable adaptability to diverse environments (Rosenthal et al. 2021). Guppies demonstrate remarkable salinity acclimation abilities, tolerating varying salt concentrations up to 35‰ and even exceeding those of seawater (Chervinski 1984, Shikano and Fujio 1998). Notably, newborn guppies are more tolerant to salinity than adults, and there have been observations of individuals successfully reproducing in seawater, leading to the establishment of seawater-tolerant strains (Shikano 1997).

Environmental salinity directly impacts various physiological processes in aquatic organisms, including osmoregulation, hormonal regulation, energy metabolism, immune responses, and growth (Laiz-Carrión 2005; Jiang et al. 2008). Several studies have investigated the effects of salt stress on guppy physiology. For instance, salinity stress has been associated with decreased levels of sex steroids in these fish (Moniruzzaman et al. 2018), while the presence of salt has been shown to mitigate lipid peroxidation (Leitemperger et al. 2019). Moreover, elevated salinity levels have been linked to increased anxiety behavior and oxidative stress (Xu et al. 2023).

The maintenance of body fluid homeostasis in fish is a highly critical process primarily associated with osmoregulatory organs such as the gills, digestive tract, and kidneys (Takei 2016). The gills are particularly vital for regulating ions and water transport (Borgatti et al. 1992; Streelman and Kocher 2002; Pavlosky et al. 2019). Recent transcriptomic analyses of gill tissues from guppies under seawater conditions revealed significant variations in gene expression associated with ion transport, cellular stress response, ATP biosynthesis, metabolic processes, and immune function, reflecting the adaptive shifts needed for osmoregulation in saltwater environments (Chen et al. 2021). The intestine plays a critical role in water and salt absorption in fish. While substantial morphological and functional alterations in the intestines of eels and medaka during adaptation to seawater have been extensively documented (Takei and Yuge 2007; Cutler and Cramb 2008; Gregorio et al. 2013; Wong et al. 2014), adaptation mechanisms in the guppy intestine during salt acclimation remain unexplored.

In this study, we investigated the adaptive responses of the intestine to salinity stress in guppies (*Poecilia reticulata*), shedding light on morphological alterations and transcriptome dynamics induced by varying salinity conditions. Morphological and transcriptome analyses revealed significant changes in the intestinal structure, including diameter and villus height, along with the expression of genes related to osmoregulation, tissue repair, and growth in saltwater-acclimated individuals. These results contribute to elucidating the mechanisms driving adaptation to varying environmental salinity in guppies.

## Materials and methods

### Fish and experimental design

Adult wild-type zebrafish and guppies were housed in a standard circulating water system at 28°C under a 14-h light/10-h dark cycle (Koun et al. 2023). In the osmotic challenge experiments, 3‰ salt was added every 2 days to separate tanks containing either guppies or zebrafish. The osmotic challenge continued until the salinity reached 35‰ for guppies or 15‰ for zebrafish over a period of 24 days. Mortality was measured for both species at each salinity level (n = 10 for guppies and n = 7 for zebrafish). All experimental procedures were conducted according to the guidelines and regulations of the Animal Care and Use Committee.

### Histological examination

Following exposure to varying salt concentrations (0‰, 18‰, and 35‰ for guppies; 0‰ and 12‰ for zebrafish), the intestine of each fish (n = 5 for each group) was dissected and fixed in 10% formaldehyde for more than 24 h at room temperature. Subsequently, it was rinsed with sterile distilled water for 10 min and dehydrated through a series of alcohol solutions at 70%, 80%, 90%, 95%, and 98% and two substitutions with 100% alcohol for 30 min at each concentration. The tissues were then cleared in a solution of alcohol and xylene (3:1, 1:1, and 1:3) for 30 min each, followed by immersion in xylene twice for 30 min each time. After deaeration in the final xylene step, the tissues were immersed in a paraffin solution (xylene:paraffin 2:1, 1:2) for 1 h and then in 100% paraffin twice for 1 h each time. Finally, the tissues were embedded in a paraffin block overnight. Sections with a thickness of 5 μm were obtained from the paraffin-embedded block using a microtome; then, they were affixed onto slide glasses at 50°C using a slide warmer and dried for 3 h. For staining, the slides were deparaffinized twice in xylene for 5 min each time, followed by sequential 5-min immersions in 100%, 95%, 70%, and 50% alcohol. The slides were then rinsed in sterile distilled water for 10 min. Subsequently, they were placed in a container with hematoxylin staining solution and incubated at room temperature for 5 min. After incubation, they were rinsed in sterile distilled water for 10 min and stained with eosin staining solution for 5 min. Once the staining was complete, they were washed with sterile distilled water and dehydrated in 70%, 80%, 90%, and 100% alcohol for 1 min at each concentration. Finally, the slides were cleansed with xylene and covered with coverslips using the mounting solution. The stained tissue sections were examined under a microscope.

### Chromatin extraction and western blotting

The intestinal tissues from guppies adapted to salt stress levels of 0‰, 18‰, and 35‰ were subjected to lysis via buffer A (10 mM HEPES at pH 7.9, 10 mM KCl, 1.5 mM MgCl_2_, 0.34 mM sucrose, 10% glycerol, 1 mM DTT, 1 mM PMSF, and 0.1% Triton X-100) using a TissueLyser LT (Qiagen). Following centrifugation for 5 min, the supernatant was removed, and the remaining pellet was dissolved in SDS sample buffer. The following antibodies were used for subsequent analyses: H3K9ac, H3K18ac, H3K9me3, and H3K36me3 from Active Motif (Carlsbad, CA, USA); H3K27ac and H3K4me3 from Abcam (LON, GBR); and H3K27me3 from Millipore (Burlington, MA, USA).

### RNA sequencing and quantitative real-time PCR (qRT-PCR)

Total RNA was extracted from the intestinal samples obtained from guppies subjected to salt stress levels of 0‰, 18‰, and 35‰. High-throughput sequencing was performed using 100-bp paired-end reads on a HiSeq 2500 platform (Illumina, CA, USA). The sequenced reads were aligned to the *P. reticulata* genome using the STAR aligner (Dobin et al. 2013). Transcript abundance was quantified using StringTie (Pertea et al. 2015), and gene expression levels were measured in transcripts per million. Differential gene expression analysis was conducted using the DESeq2 package (Love et al. 2014), with a cutoff threshold set at a fold change >1.5 and a false discovery rate (FDR) < 0.05. RNA-seq data are available under GEO accession number GSE263966. In addition, the raw transcriptome data from guppy gill tissues were downloaded from the China National GeneBank (https://ngdc.cncb.ac.cn/gsa) under accession number CRA002620. Principal component analysis (PCA) was conducted using ClustVis (https://bilt.cs.ut.ee/clustvis/), with the first two principal components (PC1 and PC2) plotted on the X and Y axes, respectively (Kim et al. 2021). K-means clustering and gene ontology (GO) analyses were performed using the Morpheus website (https://software.boradinstitute.org/morpheus/) and the Metascape tool, respectively (Lee et al. 2020). The ChEA3 tool was employed to predict transcription factors associated with a set of genes (Keenan et al. 2019). The transcription factor – gene interaction network was developed using GeneMANIA (Version 3.5.3), with zebrafish designated as the target organism for analyzing input gene interactions, including physical interactions and co-expression (Warde-Farley et al. 2010). The constructed network was visualized using Cytoscape (Version 3.10.3) to enhance the interpretability of results and their presentation.

IQ SYBR Green SuperMix (Bio-Rad, Hercules, CA, USA) was used for qRT-PCR (Bong et al. 2024). Specific primers designed for *P. reticulata* and *D. rerio* were used for amplifications. The primer sequences for *P. reticulata* are listed in Table S1.

### Chromatin immunoprecipitation (ChIP) assay

Small intestinal samples from *P. reticulata* and *D. rerio* were cross-linked using 1.5% formaldehyde for 15 min. Tissue lysates were prepared using a hypotonic buffer (10 mM HEPES-KOH at pH 7.8, 10 mM KCl, 1.5 mM MgCl_2_, 5 μM trichostatin A, and protease inhibitors) in a TissueLyser LT (Qiagen). The lysates were sonicated using a Bioruptor device (Diagenode) for 20 cycles. Following preclearing, ChIP assays were performed using antibodies specific to H3K9ac and H3K9me3. The DNA obtained from immunoprecipitation was analyzed via qRT-PCR using primers targeting specific regions of the *fosl2*, *pik3cb*, and *slc12a1* promoters. The primers used in this assay are listed in Table S1.

### Statistical analysis

Quantitative data are presented as the mean ± standard deviation (SD). Statistical analyses were conducted in GraphPad Prism 7, using one-way ANOVA followed by Tukey’s multiple comparisons test or a two-tailed *t*-test.

## Results

### Morphological changes in the guppy intestine during salinity stress

**Figure 1.**
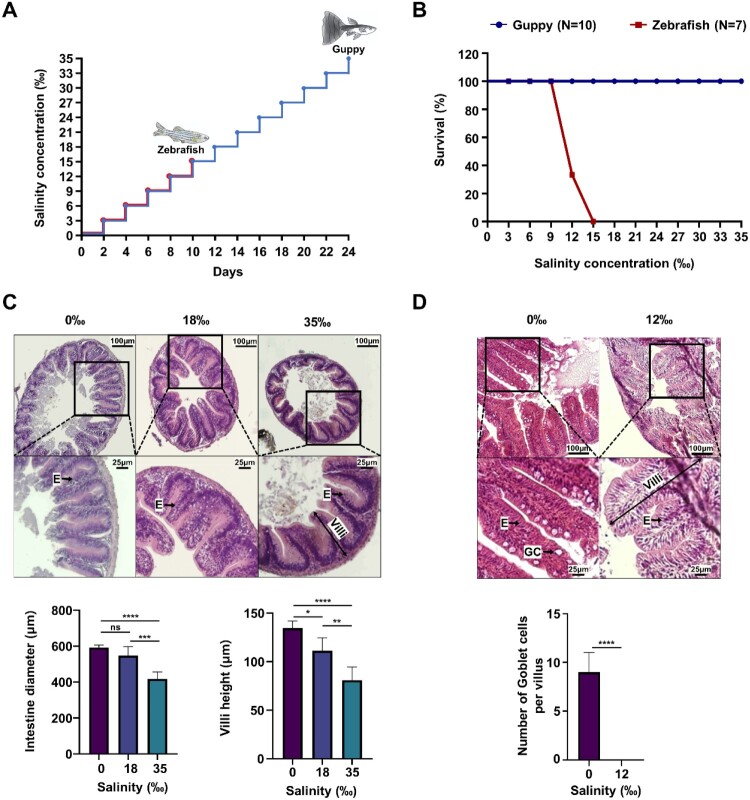
Morphological changes in the intestine of guppies and zebrafish under salinity stress. (A) Schematic design of salinity adaptation experiment for guppies and zebrafish. Salt was added to tanks every 2 days, increasing by 3‰ until the guppy tanks reached 35‰ and zebrafish tanks reached 15‰. (B) A survival rate graph was plotted against varying salinity levels, with mortalities documented for both guppies and zebrafish at each level (n = 10 for guppies, n = 7 for zebrafish). (C) Images of H & E stained sections of the middle part of small intestine sections from the guppy adapted to the three salt conditions (0‰,18‰, and 35‰) were taken at 100x magnification using a microscope. The lower panels show zoomed-in views of the areas outlined black squares. The intestine diameter and villus height were measured, and the results are shown as the mean ± SD. *P* value is determined by one-way ANOVA, Tukey's multiple comparisons test. ns, not significant; *P < 0.05; **P < 0.01; ***P < 0.001; ****P < 0.0001. (D) H & E stained sections of the middle part of small intestine from zebrafish adapted to the two salt conditions (0‰ and 12‰). The lower panels show magnified views of the areas outlined by black squares. The number of goblet cells was counted, and the data are presented as the mean ± SEM. *P* value is determined by Two-tailed t-test. ****P < 0.0001. E, enterocyte; GC, goblet cell.

Guppies are euryhaline fish known for their adaptability to a wide range of salinity changes. To investigate the molecular mechanisms underlying their high salt tolerance, we designed a salinity adaptation experiment using zebrafish – a stenohaline species with limited salinity tolerance – as a control group. In this experiment, salinity was gradually increased by 3‰ up to 35‰ (Figure 1A). This adaptation protocol was used to initially assess the survival rate of guppies under varying salinity levels. As expected, individuals adapted well to the 35‰ salinity and survived without manifesting abnormalities such as the refusal to eat, loss of balance, or shortness of breath (Figure 1B and Supplementary Video files S1 and S2). In contrast, the zebrafish survived up to a salinity of 9‰, but they became noticeably slower, and their feeding activity was significantly reduced and unbalanced when the level reached 12‰. Finally, at 15‰, all individuals died (Supplementary Video files S3 and S4). These observations confirm that euryhaline fish, such as guppies, have a greater salt tolerance than stenohaline fish, like zebrafish, indicating that our salt adaptation experiment was adequately designed and properly performed.

The gills are the primary organ that regulates osmotic pressure through ion exchange in fish, but other organs, such as the intestine and the kidneys, also perform this function (Scott et al. 2006; Takei and Yuge 2007; Salati 2011; Martinez 2012). In particular, in euryhaline fish, the intestine plays a critical role in osmoregulation and the ability to avoid dehydration. Therefore, this study sought to examine whether salt stress affects the morphology and function of the intestine in guppies. After the fish were adapted to three salt concentrations (0‰, 18‰, and 35‰), their small intestine was dissected and fixed in formaldehyde for 24 h. Then, after obtaining paraffin-embedded sections, the tissues were subjected to hematoxylin and eosin staining. The results showed that, in guppies, as salinity increased, both villus height and intestinal diameter decreased (Figure 1C). This reduction in surface area may represent an adaptive mechanism to cope with high salinity, potentially aimed at minimizing salt absorption by reducing the overall absorptive area of the intestine (Su et al. 2022). Additionally, the analysis revealed a higher density of columnar-shaped enterocytes, likely resulting from the reduction in villus height and intestinal diameter. Despite these structural changes, the villus structure remained intact, suggesting that guppies maintain their absorption function while modulating their intestinal structure to cope with elevated salt levels. In contrast, in zebrafish exposed to 12‰ salinity, significant damage to the intestine was observed, including the disappearance of goblet cells and a severely damaged villus structure (Figure 1D). These differences between the two species suggest that guppies adapt to changes in salinity by strategically reducing their intestinal surface area to control salt absorption, whereas zebrafish are more susceptible to damage, which ultimately compromises their intestinal function.

### Transcriptome dynamics in the intestine of guppies under salinity stress

**Figure 2.**
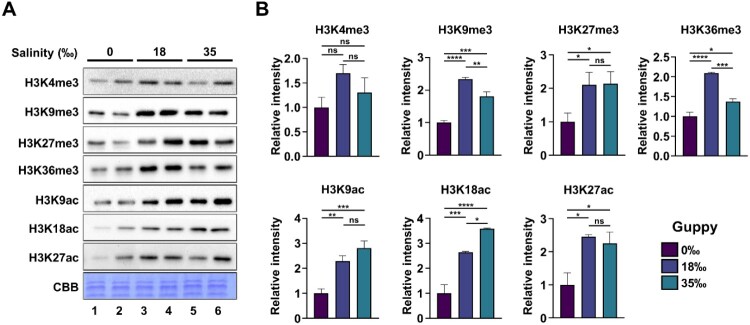
Dynamic histone modifications in response to salinity stress. (A) Histones were extracted from the guppy intestine adapted to the three salt conditions (0‰,18‰, and 35‰), followed by an analysis of post-translational modifications using the indicated antibodies through Western blotting. CBB, coomassie brilliant blue staining. (B) The band intensity as in (A) was quantified using Image J software. The value from 0‰ was set to 1, and the relative intensities of other lanes were expressed accordingly. The data are presented as the mean ± SEM. *P* value is determined by one-way ANOVA, Dunnett's multiple comparisons test. ns, not significant; *P < 0.05; **P < 0.01; ***P < 0.001; ****P < 0.0001.

Histone modification plays an important role in stress tolerance by regulating gene expression (Li et al. 2017; Yang et al. 2022; Mojica 2023). To examine the effect of salinity stress on histone modifications, chromatin fractions in intestinal tissues obtained from guppies exposed to different salt concentrations were analyzed by western blotting as indicated antibodies. The levels of H3K9ac and H3K18ac acetylation gradually increased in a concentration-dependent manner. H3K27ac levels peaked at 18‰ and remained elevated at 35‰. In contrast, the overall levels of H3 methylation such as H3K9 and H3K36 increased at 18‰ and then decreased at 35‰ (Figure 2). H3K27me3 levels reached their highest point at 18‰ and remained elevated at 35‰. These findings suggest that dynamic histone modifications play a role in regulating gene expression in response to salinity stress in *P. reticulata*.

**Figure 3.**
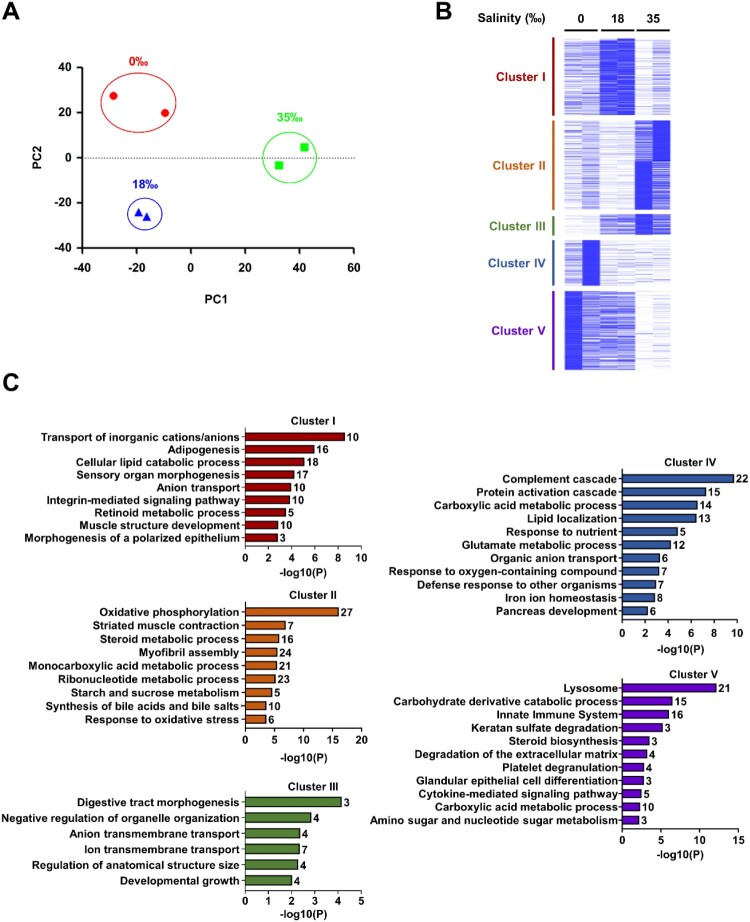
Dynamic gene expression profile and transcriptional features in intestine under salinity stress. (A) Principal component analysis (PCA) plot of gene expression data from three salt stress conditions at the guppy intestine. The PCA plot shows a clear separation between three salt conditions 0‰ (red), 18‰ (blue), and 35‰ (green) samples. (B) K-means clustering with K = 5 was utilized to cluster differentially expressed genes from pairwise comparisons among the three conditions. (C) Gene ontology analysis was conducted for each clustered group. The number next to the bar represents the count of genes in the GO term.

Given the potential correlations among morphological, histone, and transcriptional modifications, RNA-sequencing was employed to specifically investigate the latter in the intestine of guppies exposed to different salinity stress levels (i.e. 0‰, 18‰, and 35‰). After mapping the obtained sequences to the reference genome (*P. reticulata*), a PCA plot was generated to analyze the heterogeneity of transcriptome data. It was revealed that the data for tissues exposed to 35‰ salinity were distinctly separated from those obtained for tissues under the two other salinity conditions (Figure 3A). The analysis of RNA-seq data revealed 691 differentially expressed genes in any pairwise comparison of the three conditions. K-means cluster analysis identified five gene clusters (Figure 3B and Table S2). GO analysis was then conducted to gain insights into the biological functions of these clusters, revealing specific terms associated with each of them (Figure 3C). Cluster I, characterized by genes upregulated at 18‰ salinity, was enriched for functions related to the transport of inorganic ions and amino acids, suggesting adaptive mechanisms for maintaining ion homeostasis. Cluster II, showing upregulation at 35‰, was associated with oxidative phosphorylation, indicating enhanced energy production under high salinity. Cluster III, which exhibited a salinity-dependent increase in expression, was enriched for terms related to digestive tract morphogenesis, developmental growth, and ion transmembrane transport, highlighting its potential role in structural and functional adaptation. Cluster IV, upregulated at 0‰, was linked to the complement cascade, implicating an active immune response under freshwater conditions. Lastly, Cluster V, showing a gradual decrease in gene expression with increasing salinity, was enriched for lysosome and innate immune system functions, suggesting a reduced reliance on innate immune processes under high salinity.

### Osmoregulation in the intestine of guppies in response to salinity stress

Recent studies have shown that euryhaline fish reorganize the gill and intestinal epithelia to meet the requirements of ion transport and permeability (Hirai 1999; Carmona 2004; Souza 2007; Guo et al. 2018; Bonzi et al. 2021).

**Figure 4.**
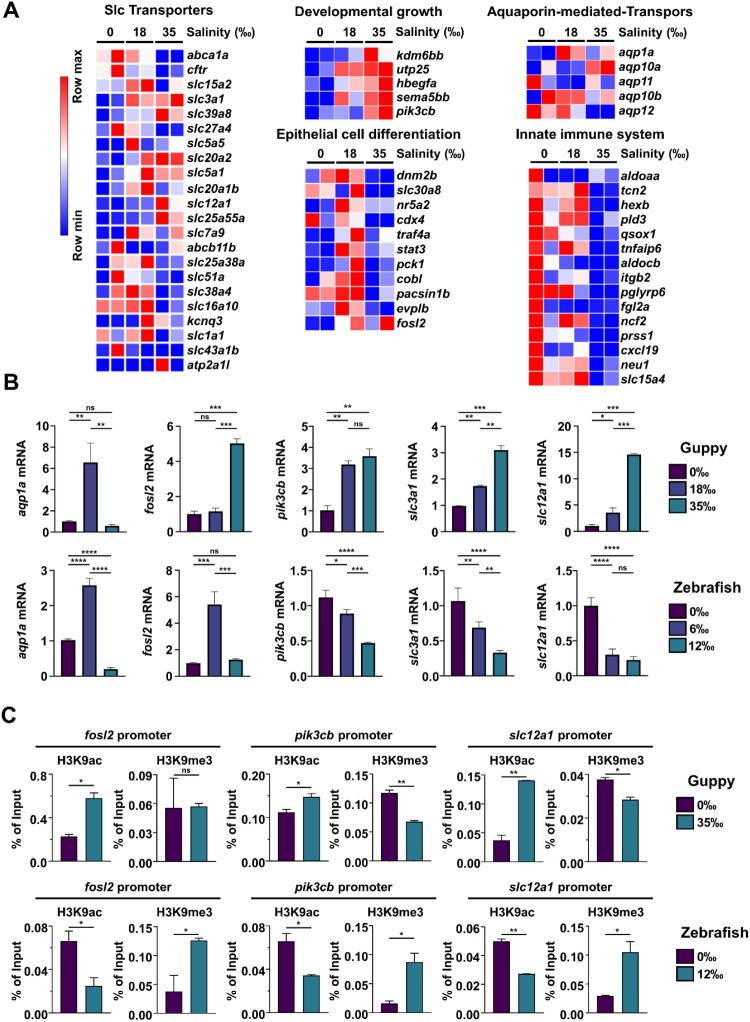
Osmoregulatory system in the guppy intestine in response to salinity stress. (A) Heatmap showing the transcripts per million of DEGs involved in osmoregulation. (B) RNA was isolated from guppies or zebrafish adapted to various salt conditions, followed by qRT-PCR analysis using the specified primers. The data are shown as the mean ± SD. *P* value is determined by one-way ANOVA, Tukey's multiple comparisons test. ns, not significant; *P < 0.05; **P < 0.01; ***P < 0.001; ****P < 0.0001. (C) ChIP assays of fosl2, pik3cb, and slc12a1 genes were performed using anti-H3K9ac and anti-H3K9me3 antibodies in the small intestines of guppies and zebrafish as in (B). The data are presented as the mean ± SD. *P* value is determined by Two-tailed *t*-test. ns, not significant; *P < 0.05; **P < 0.01.

**Figure 5.**
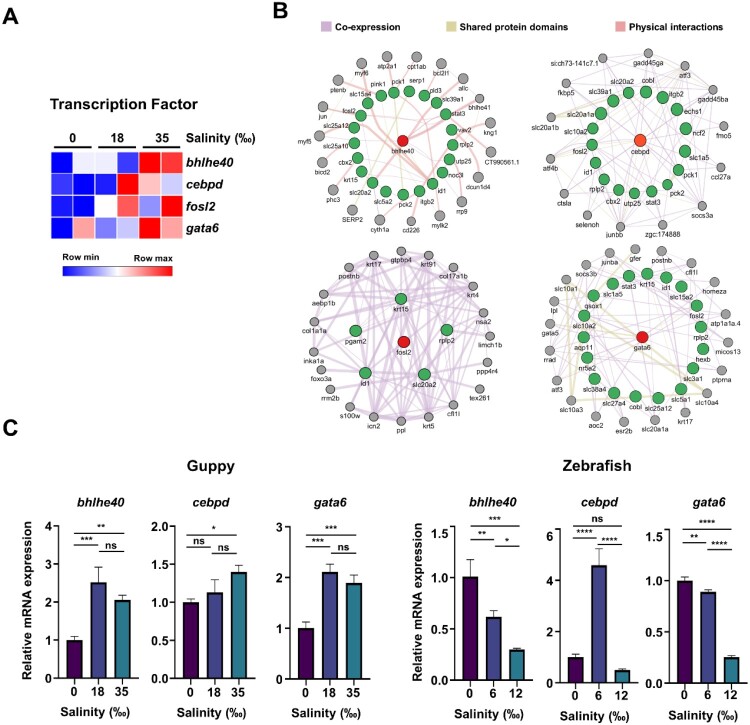
Putative transcription factors in salinity stress adaptation. (A) Prediction of transcription factors regulating the expression of genes belonging to cluster III, as in Figure 3B, using ChEA3. (B) Transcription factor-target gene interaction network from (A) was constructed using GeneMANIA and visualized in Cytoscape. The line color represents the types of network interaction. Red, transcription factor; green, target genes from ChEA3; grey, target genes from GeneMANIA. (C) qRT-PCR analysis of the RNA isolated from guppies (0‰,18‰, and 35‰) or zebrafish (0‰, 6‰, and 12‰) as in Figure 4B. The data are shown as the mean ± SD. *P* value is determined by one-way ANOVA. ns, not significant; *P < 0.05; **P < 0.01; ***P < 0.001; ****P < 0.0001.

Consistent with previous research, the histological and transcriptomic data obtained in the present study demonstrated that the intestinal enterocytes of guppies changed morphologically and functionally in response to salinity stress. RNA-seq data indicated a substantial upregulation of osmoregulatory genes associated with epithelial cell differentiation, developmental growth, and the transport of ions and water molecules. Concurrently, genes related to the innate immune system were shown to be downregulated (Figure 4A); a finding that was corroborated by qRT-PCR analysis (Figure 4B). On the other hand, in zebrafish, the expression of some genes (such as *slc3a1*, *slc12a1*, and *Pik3cb*) decreased under salt concentrations of 6‰ and 12‰. Next, we studied the mechanism by which high salinity increased the expression of adaptation genes (such as the *fosl2*, *slc12a1,* and *pik3b*) in guppies but not in zebrafish. To this end, ChIP assays were conducted to investigate the epigenetic changes in the promoter regions of genes related to salt adaptation. As expected, our results showed that H3K9ac levels increased and H3K9me3 levels decreased in guppies exposed to 35‰ salinity. Conversely, in zebrafish, H3K9me3 levels gradually increased under similar conditions (Figure 4C). Finally, the bioinformatics tool ChEA3 was employed to identify transcription factors that regulate those genes that undergo upregulation under salt stress. This analysis specifically focused on salt stress-related transcription factors whose expression levels increased in response to high salinity. Notably, transcription factors such as *bhlhe40*, *cebpd*, and *gata6* were found to be progressively upregulated in guppies as salinity increased from 0‰ to 18‰ and then up to 35‰ (Figure 5A). A network analysis of the relationship between these transcription factors and their target genes revealed a complex regulatory network (Figure 5B). In contrast, in zebrafish, the expression of *bhlhe40* and *gata6* decreased, while that of *cebpd* initially increased at 6‰ but then decreased at 12‰ (Figures 5C).

These findings suggest that the differential expression of salt adaptation-related genes between guppies and zebrafish could be due to variations in both epigenetic modifications and the activity of specific transcription factors under high salinity.

## Discussion

The present study aimed to unravel the intricate mechanisms underlying the adaptation of guppies to varying salinity conditions. Our experimental approach involved subjecting the fish to a stepwise increase in salinity up to 35‰, and the results revealed their remarkable salt tolerance compared with zebrafish. The focus was on the crucial role of the intestine in osmoregulation; a function that is traditionally associated with the gills. Morphological analysis of the intestinal tissues of guppies adapted to different salinity conditions revealed significant changes in the size of the intestine and villi, along with the presence of condensed columnar-shaped enterocytes, indicating dynamic adaptations to maintain osmotic balance.

### Critical genes in osmoregulation

In the intricate response of the guppy intestine to salinity stress, several key genes were identified as crucial players orchestrating adaptive osmoregulation. Notably, the upregulation of the SLC transporters is indicative of their pivotal roles in maintaining ion homeostasis under high salinity (Cutler and Cramb 2008; Gregorio et al. 2013; Wong et al. 2014). Slc12a1, also known as the Na-K-Cl cotransporter, is instrumental in facilitating the transport of sodium, potassium, and chloride ions across cell membranes. In addition, this transporter may operate alongside bicarbonate secretion to enhance water absorption, facilitating efficient osmoregulation in high-salinity environments (Esbaugh and Cutler 2016). Interestingly, the expression of *slc12a1* in the intestine was significantly reduced in zebrafish exposed to high salinity, while it was markedly increased in guppies under the same conditions. This species-specific upregulation likely reflects an adaptive mechanism to cope with prolonged salt stress, supporting the critical ion transport processes required to maintain homeostasis in challenging osmotic conditions. Similarly, the guppy intestine exhibited a significant increase in the expression of *slc3a1*, a component of the heterodimeric amino acid transporter, and *aqp1a*, a water channel. During osmotic acclimation, euryhaline fish exhibit increased energy demands, and amino acids serve as energy substrates and osmolytes in both plasma and cells at this time (Sundell et al. 2019). The upregulation of *Slc3a1* may play a crucial role in energy metabolism and osmoregulation. Interestingly, the upregulation of *aqp1a* in the guppy intestine during salinity acclimation mirrors that observed in the eel intestine (Aoki et al. 2003; Martinez et al. 2005). However, other euryhaline fish species such as sea bream and medaka have been reported to show decreased expression of *aqp1* during salinity stress (An et al. 2008; Madsen et al. 2014; Wong et al. 2014). Considering the high demand for intestinal water absorption in seawater teleosts, additional investigations are warranted to elucidate the underlying reasons for the variability in *aqp1* expression levels among these species. These studies may shed light on the regulatory factors influencing *aqp1* expression across diverse environmental conditions.

### Genes controlling tissue remodeling for osmoregulation

Genes associated with epithelial cell differentiation, such as *fosl2*, exhibit increased expression levels in response to salinity stress. Fosl2, which belongs to the Fos transcription factor family, is implicated in the regulation of diverse cellular processes, including proliferation, differentiation, and apoptosis (Bozec et al. 2013; Li et al. 2022; Shetty et al. 2022). Its upregulation suggests a potential role in modulating the structural integrity and barrier function of the intestinal epithelium in guppies subjected to salinity fluctuations. By promoting epithelial cell differentiation, Fosl2 may contribute to fortifying the intestinal barrier, thereby mitigating water loss and preserving osmotic equilibrium in response to heightened salinity. In addition, genes involved in developmental growth, such as *pik3cb*, demonstrate heightened expression levels in the intestine of guppies under salinity stress. Specifically, *pik3cb* encodes the catalytic subunit of phosphoinositide 3-kinase (PI3 K), a pivotal signaling enzyme implicated in various cellular processes, including growth, proliferation, and survival (Lin et al. 2015; Yang et al. 2019). Its upregulation suggests an adaptive growth response aimed at bolstering tissue resilience and functional capacity in the face of salinity-induced stress. By promoting cellular growth and proliferation, *Pik3cb* may facilitate tissue repair and remodeling, thereby contributing to the overall adaptive response of the guppy intestine to salinity stress.

### Role of innate immunity genes

In addition, to the upregulation of genes involved in osmoregulation and tissue remodeling, our study revealed the downregulation of genes associated with the innate immune system in the intestine of guppies under salinity stress. Specifically, *tnfaip6*, *itgb2*, and *pglyrp6* stand out as key genes showing reduced expression levels in response to elevated salinity. These genes play critical roles in inflammation (*tnfaip6*), immune cell adhesion and migration (*itgb2*) as well as in the recognition of bacteria and initiation of immune responses (*pglyrp6*) (Chang and Zhang 2017; Wei et al. 2021 Li et al. 2022;). The downregulation of genes associated with the innate immune system suggests a potential trade-off mechanism wherein resources are reallocated from immune defense toward processes that are crucial for coping with salinity stress. This trade-off strategy aligns with the concept of resource allocation theory, according to which organisms prioritize resources toward functions essential for immediate survival or reproduction when faced with environmental stressors (Reznick 2002). In the context of the guppy intestine, the observed downregulation of immune-related genes may indicate a prioritization of resources toward maintaining osmoregulatory balance and tissue remodeling to withstand salinity fluctuations.

### Epigenetic modifications and transcription factors involved in salt stress adaptation

Epigenetic modifications, particularly histone acetylation and methylation, are crucial in regulating gene expression in response to environmental changes, including salt stress (Jaenisch and Bird 2003; Rashid et al. 2022). In our study, significant histone modifications were observed within the intestine of guppies under salt stress, which highlights the role of epigenetic mechanisms in adaptive responses. Specifically, our findings revealed a global increase in histone acetylation marks, such as H3K9ac and H3K18ac, both of which are typically associated with transcriptional activation. Notably, the ChIP assay showed elevated levels of H3K9ac in guppies exposed to high salinity, suggesting that this epigenetic modification plays a key role in the transcriptional activation of genes involved in osmoregulation and tissue remodeling. In contrast, histone methylation exhibited a more complex response to salt stress. Methylation marks associated with both gene activation (H3K4me3 and H3K36me3) and repression (H3K9me3 and H3K27me3) showed intricate patterns. The analysis revealed an increase in the global levels of repressive marks, i.e. H3K9me3 and H3K27me3, at higher salinity levels (18‰ and 35‰). Interestingly, the H3K9me3 levels increased globally but decreased specifically in the promoter regions of osmoregulatory genes at 35‰. This reduction in H3K9me3 in key promoter regions corresponded with the upregulation of osmoregulatory genes, emphasizing the importance of targeted histone demethylation in facilitating the adaptive transcriptional response to salt stress. In contrast, zebrafish exhibited histone modifications in the promoter regions of salt stress-related genes that were opposite to those observed in guppies. This difference in epigenetic responses may explain the reduced ability of zebrafish to adapt to high-salinity environments.

The interplay between epigenetic modifications and transcription factors is critical in orchestrating the gene expression changes necessary for salt adaptation. Our transcriptomic analysis revealed that, in response to increasing salinity, several transcription factors, including *bhlhe40*, *cebpd*, *fosl2*, and *gata6*, were upregulated in guppies. These factors are known to be involved in stress response pathways, tissue remodeling, and cellular growth, all of which are essential for maintaining osmotic balance in hyperosmotic environments (Acosta-Iborra et al. 2024, Balamurugan and Sterneck 2013, Decker et al. 2006, Li et al. 2022).

Bhlhe40 is involved in circadian rhythms and hypoxic stress responses (Wang et al. 2015; Kiss et al. 2020). Based on the results of network analysis, Bhlhe40 is expected to regulate *slc39a1*, a transporter involved in zinc homeostasis (Takagishi et al. 2017), and *slc5a2*, which contributes to ionic balance (Jiang et al. 2014). Additionally, it is probably involved in mitochondrial function and ion transport by upregulating *slc25a10* and *slc25a12*, which are important for mitochondrial activity and cellular homeostasis (Cai et al. 2019). This transcription factor may also interact with STAT3 (John et al. 2019), a key player in inflammatory signaling and cell survival, further supporting cellular resilience under stress.

Cebpd, a member of the CCAAT/enhancer-binding protein family, plays a central role in various cellular processes, including differentiation, metabolism, and immune responses (Cardinaux and Magistretti 1996; Tanaka et al. 1997; Zannetti et al. 2010). PPI network analysis suggested that Cebpd may upregulate *slc39a1*, a zinc transporter, which could enhance antioxidant defense by facilitating zinc transport (Jeong and Eide 2013). Additionally, Cebpd is likely to regulate *ncf2*, a subunit of NADPH oxidase, which assists in managing oxidative stress by modulating the production of reactive oxygen species (Ngo and Duennwald 2022). Moreover, Cebpd is expected to influence ion homeostasis by regulating transporters such as *slc10a2* and *slc20a2*, which are involved in phosphate and bile acid transport (Hruz et al. 2006; Ramos-Brossier et al. 2024), respectively. This transcription factor may also support energy balance under stress conditions by activating key metabolic genes such as *pck1* (gluconeogenesis) and *rplp2* (ribosomal protein synthesis) (Artero-Castro et al. 2015; Verissimo et al. 2023). Collectively, these functions suggest that Cebpd supports cellular survival under salt stress by regulating ion transport, oxidative stress responses, and energy homeostasis.

Fosl2, a member of the AP-1 complex, modulates cellular proliferation and differentiation (Jahangiri et al. 2016). In response to salt stress, Fosl2 may upregulate genes such as *slc20a2* and *krt15 (*Ren et al. 2021*;* Ievlev et al. 2023*)*, which help maintain cell integrity under stress conditions. It can also regulate *pgam2*, an enzyme involved in glycolysis (Okuda et al. 2013), to support energy production during salt-induced stress. Additionally, Fosl2 may influence genes involved in cell growth and protein synthesis, such as *id1*, *rplp2*, and *arap1 (*Sikder et al. 2003*;* Artero-Castro et al. 2015*;* Zhang et al. 2023*)*, which are essential for cell survival and proper function in hyperosmotic environments.

Gata6, a transcription factor essential for cellular development and differentiation (Koutsourakis et al. 1999; Keijzer et al. 2001), plays a pivotal role in the cellular response to salt stress. It is expected to regulate genes related to nutrient and ion transport, such as *slc15a2*, *slc3a1*, and *slc1a5 (*Zhang et al. 2019*;* Nwosu et al. 2023*)*, which help maintain cellular ion balance and nutrient uptake. Additionally, Gata6 is likely to activate *gstm4*, a gene involved in oxidative stress defense (Allocati et al. 2018), which assists in protecting cells from oxidative damage induced by salt stress. Furthermore, Gata6 may regulate stress-responsive genes such as *stat3* and *fosl2*, both of which are critical for maintaining cellular survival and function under challenging conditions. These functions suggest that Gata6 plays an important role in cellular resilience during salt-induced stress, supporting ion balance, oxidative stress responses, and cell survival mechanisms (Rong et al. 2012; Toyama et al. 2023).

Collectively, these four transcription factors – *bhlhe40*, *cebpd*, *fosl2*, and *gata6* – work in concert to regulate complex gene expression processes that enable cells to adapt to salt stress. Their roles in ion transport, oxidative stress management, and metabolic processes highlight their critical involvement in cellular resilience and in the mechanisms of adaptation to stress.

Interestingly, when compared with guppies, zebrafish, which exhibit a lower salt tolerance, showed a different transcriptional response to salt stress. In zebrafish, the expression of *bhlhe40* and *gata6* decreased under high salinity, whereas that of *cebpd* transiently increased at lower salinity and declined at higher salinity.

The decrease in *bhlhe40* and *gata6* expression in zebrafish could reflect an impaired ability to regulate essential stress response pathways, including ion balance, oxidative stress mitigation, and cellular survival under osmotic pressure. The transient increase followed by a decline in *cebpd* expression suggests a temporary cellular stress response that cannot be sustained as salinity increases, possibly due to the adaptation mechanism becoming overwhelmed. This divergent transcriptional response is likely associated with the different abilities of these species to cope with hyperosmotic conditions, with zebrafish being less able to maintain homeostasis at high salinity. These findings suggest that the differences in transcriptional regulation between species, for example in terms of the modulation of *bhlhe40*, *cebpd*, and *gata6*, may explain their respective salt tolerance levels. However, further experimental validation is needed to confirm these potential regulatory mechanisms in vivo and to explore how epigenetic modifications and transcriptional networks are integrated to support cellular resilience in response to salt stress.

## Comparative analysis of the transcriptome in intestinal and gill tissues under salt stress

The intestine and gills of guppies under salt stress exhibited complementary yet distinct roles in maintaining osmoregulatory homeostasis, with organ-specific transcriptional responses. The gills, as a multifunctional organ, primarily facilitate gas exchange, ion secretion, and nitrogenous waste excretion, while contributing to acid–base balance and immune defense (Evans et al. 2005). In contrast, the intestine is specialized in ion absorption, water uptake, and nutrient digestion, which makes it an essential organ for hydration and energy acquisition in marine environments (Whittamore 2012). The comparative analysis of gene expression patterns in these tissues further corroborated the above-mentioned organ-specific roles (Table S3).

The observed upregulation of *slc12a1* and other *slc* transporters in the intestine emphasizes the role of this organ in ion absorption and water uptake, which are critical for osmoregulation. The increased expression of genes related to tissue remodeling and growth, such as *pi3cb* and *fosl2*, indicated structural adaptation to sustained stress. In addition, the intestine exhibited increased expression of specific transcription factors like *cebpd*, *fosl2*, and *gata6*, which play critical roles in cellular remodeling and adaptive responses to stress.

In contrast, the gills showed higher expression of *slc12a2* and *cftr*, which facilitate ion secretion, underscoring the role of this organ in expelling excess salts to maintain osmotic balance (Table S3). Growth-related genes in the gills either remained unaltered or were downregulated, reflecting a reliance on functional rather than structural adjustments. Notably, transcription factors like *cebpd* and *fosl2* exhibited upregulation in the intestine but either decreased expression or no significant change in the gills. This suggests that different transcriptional regulators may be involved in the response of the gills to salt stress, underscoring the organ-specific nature of these regulatory mechanisms. Both in the intestine and gills, oxidative phosphorylation-related genes were upregulated, which reflects the high energy demands of osmoregulation. However, immune-related genes were consistently downregulated in both organs, suggesting a trade-off that prioritizes osmoregulatory functions over immune responses. These findings elucidate the complementary strategies adopted by the gills and intestine in guppies: the former organ primarily manages salt excretion and gas exchange, while the latter adapts structurally to optimize ion absorption and hydration under challenging environmental conditions.

## Conclusions

Our study elucidates the remarkable adaptive capabilities of guppies (*P. reticulata*) in response to salinity stress, highlighting the critical role of the intestine as an osmoregulatory organ. Unlike zebrafish, guppies exhibit significant morphological and transcriptional changes that enable efficient osmotic balance in hyperosmotic environments. The upregulation of key osmoregulatory genes, such as *slc12a1, slc3a1,* and *aqp1a*, combined with the structural remodeling of the intestinal epithelium, reflects a dynamic strategy to cope with salt stress. In addition, genes regulating tissue remodeling, such as f*osl2* and *pik3cb*, contribute to maintaining epithelial integrity and resilience under prolonged salinity challenges. The observed epigenetic modifications, including histone acetylation and targeted demethylation, provide insights into the regulatory mechanisms facilitating these adaptive responses. The interplay of transcription factors like *bhlhe40*, *cebpd*, *fosl2*, and *gata6* is at the core of the complex regulatory networks that orchestrate cellular and physiological adaptations. Comparative transcriptomic analyses of the intestinal and gill tissues reveal complementary roles. The intestine performs functions related to ion absorption and hydration, while the gills manage ion excretion, collectively maintaining homeostasis. The findings of this study also suggest a resource allocation trade-off, with the downregulation of immune-related genes in favor of osmoregulatory and structural adaptations. This strategy prioritizes immediate survival mechanisms over immune defense during environmental stress.

In summary, guppies demonstrate a robust and multi-faceted adaptive response to salinity stress driven by transcriptional regulation, epigenetic modulation, and organ-specific functional specialization. These insights enhance our understanding of the physiological plasticity underlying euryhalinity in teleosts, providing a foundation for further studies into the evolutionary and ecological implications of osmoregulatory mechanisms.

## Limitations and future directions

This study is constrained by its focus on selected target genes and transcription factors, which limits the broader understanding of osmoregulatory networks. Future investigations should conduct comparative transcriptomic analyses of guppies and zebrafish under varying salinity conditions, capturing genome-wide transcriptional changes. Additionally, examining the roles of other transcription factors and their interactions with downstream signaling pathways could elucidate the regulatory frameworks underlying osmoregulation. Expanding this research to include proteomic and epigenomic studies would provide a more comprehensive understanding of adaptive mechanisms across species with varying salinity tolerances.

## Supplementary Material

Supplemental Material

## Data Availability

All necessary data to support the conclusions drawn in the paper are provided either within the paper or in the Supplementary information. RNA-seq data generated during this study are available under GEO accession number.
