# Supplementary figures and images for: Adaptation responses to salt stress in the gut of Poecilia reticulata
Source: Anim Cells Syst (Seoul). 2025 Jan 18;29(1):84–99. doi: 10.1080/19768354.2025.2451413 (PMC11749108; doi:10.1080/19768354.2025.2451413)

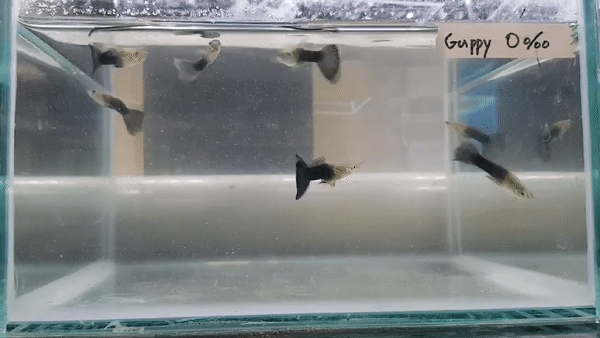

Supplement: Supplemental Material [file TACS_A_2451413_SM9521.zip › Video file S1_guppy.gif]

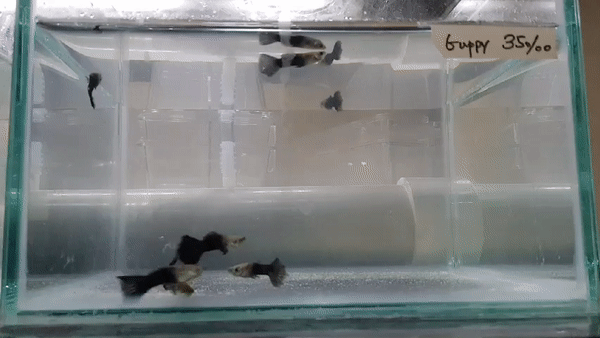

Supplement: Supplemental Material [file TACS_A_2451413_SM9521.zip › Video file S2_guppy.gif]

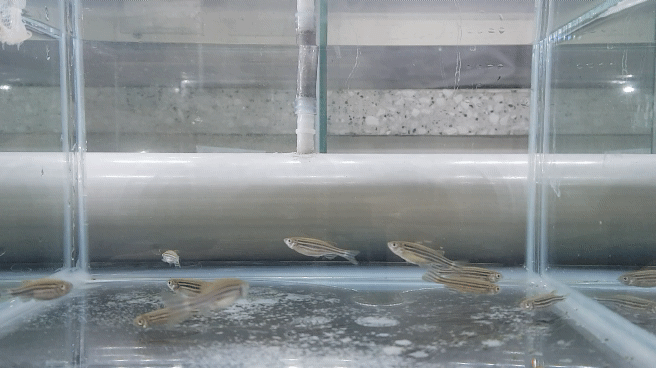

Supplement: Supplemental Material [file TACS_A_2451413_SM9521.zip › Video file S3_zebrafish.gif]

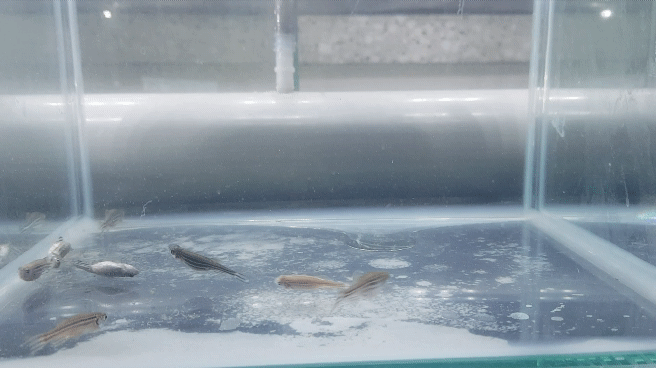

Supplement: Supplemental Material [file TACS_A_2451413_SM9521.zip › Video file S4_Zebrafish.gif]
